# Refractive error change and vision improvement in moderate to severe hyperopic amblyopia after spectacle correction: Restarting the emmetropization process?

**DOI:** 10.1371/journal.pone.0175780

**Published:** 2017-04-19

**Authors:** Ji Woong Chang

**Affiliations:** 1 Department of Ophthalmology, Inje University Ilsan Paik Hospital, Inje University College of Medicine, Goyang, Korea; 2 Graduate Program in Cognitive Science, Yonsei University, Seoul, Korea; Soochow University Medical College, CHINA

## Abstract

**Purpose:**

The aims of the study were to develop guidelines for prescribing spectacles for patients with moderate to severe hyperopic amblyopia and to demonstrate how emmetropization progresses.

**Methods:**

Children with hyperopic amblyopia who had a spherical equivalent of ≥ +4.0 diopters (D) or more were included, while those who had astigmatism of > 2.0 D or anisometropia of > 2.0 D were excluded. The patients were divided into a full correction group and an under-correction group according to the amount of hyperopia correction applied. The under-correction group was further subdivided into a fixed under-correction group and a post-cycloplegic refraction (PCR) under-correction group. The duration of amblyopia treatment and changes in initial hyperopia were compared between the groups.

**Results:**

In total, 76 eyes of 38 patients were analyzed in this study. The full correction group and under-correction group were subjected to 5.5 months and 5.9 months of amblyopia treatment, respectively (P = 0.570). However, the PCR under-correction group showed more rapid improvement (2.9 months; P = 0.001). In the under-correction group, initial hyperopia was decreased by -0.28 D and -0.49 D at 6 months and 12 months, respectively, after initial cycloplegic refraction. Moreover, the amount of hyperopia under-correction was correlated with the amount of hyperopia reduction (P = 0.010).

**Conclusion:**

The under-correction of moderate to severe hyperopic amblyopia has beneficial effects for treating amblyopia and activating emmetropization. PCR under-correction can more rapidly improve visual acuity, while both fixed under-correction and PCR under-correction can induce emmetropization and effectively reduce initial hyperopia.

## Introduction

Prescribing spectacles for hyperopia is challenging for most pediatric ophthalmologists and optometrists. When spectacles are prescribed for children with hyperopic amblyopia, three aspects of the spectacles should be considered. First, one must assess how quickly and successfully the spectacle correction can achieve corrected visual acuity. Second, one should determine whether there is any possibility of developing strabismus. Finally, one should evaluate whether the hyperopia will change in the future, which is related to the issue of emmetropization.

Hyperopia is correlated with amblyopia and strabismus. Atkinson et al. [1] demonstrated that children with more than moderate hyperopia have a greater risk of developing amblyopia and strabismus. The authors also reported that partial spectacle correction of hyperopia can reduce the risk ratios of amblyopia and strabismus [1]. However, Ingram et al. [2] investigated the effect of early spectacle correction of hyperopia at 6 months of age and reported that the incidences of strabismus and amblyopia were not reduced by early spectacle correction [2]. Atkinson et al. [1] explained that the difference might be due to differences in the methods for prescribing spectacles or in compliance with the treatment. However, Ingram et al. [3] suggested that the children with hyperopia and who later developed strabismus could not recognize a stimulus of blurred vision because of a congenital lesion. Therefore, spectacle correction could not change the development of strabismus in these children.

Spectacle correction of hyperopia is related to emmetropization, which refers to changes in neonatal refractive errors during eye growth [4]. Although emmetropization has been hypothesized to progress passively with the growth of the ocular components [5], evidence indicates that active emmetropization process is regulated by an active visual feedback control system to compensate for detected refractive errors [6–9]. Human infants usually exhibit hyperopia, after which, ocular development towards emmetropia occurs [10,11]. However, Morgan et al. [12] have argued that the preferred final endpoint of emmetropization is mild hyperopia rather than emmetropia because in areas with a low prevalence of myopia, populations tend to remain predominantly mildly hyperopic. They proved that the early onset of emmetropia is a major risk factor for myopia progression. Therefore, after the achievement of emmetropia, there is the potential for progression to myopia [12–14].

Optical blurring and accommodation induced by hyperopic defocusing promote emmetropization [4]. Atkinson et al. [15] reported that partial spectacle correction for infantile hyperopia does not impede emmetropization. However, Ingram et al. [3] demonstrated that decreases in hyperopia are impeded by partial spectacle correction for hyperopia. Emmetropization between 3 months and 12 or 24 months of age occurs under active visual feedback control and through coordinated changes in corneal power and axial length. During that period, the normal distribution of refractive errors at birth changes to a highly peaked, leptokurtic distribution, with mean values that are mild hyperopic [12,16–19]. However, several investigators have suggested that emmetropization may continue throughout life, although little evidence supports this idea [20–22]. Little is known about how emmetropization in childhood hyperopia progresses and how it is influenced by spectacle correction.

The present study thus examined hyperopia changes according to the method of spectacle correction. The aim of this study was to provide guidelines for prescribing spectacles for moderate to severe hyperopia and thus for safely achieving emmetropization progress.

## Materials and methods

This study was approved by the Inje University Ilsan Paik Hospital Institutional Review Board and was conducted in accordance with the Declaration of Helsinki ethical principles for medical research. Detailed medical records for all patients who were diagnosed as having hyperopic amblyopia between 2010 and 2015 were reviewed retrospectively. All medical records were completely anonymized, de-identified and aggregated before access to analyze the data. The patients between the age of 3 years and 11 years whose visual acuity could be measured with a Snellen acuity chart were included. Full ophthalmic evaluations at initial presentation included visual acuity measurement, slit-lamp examination of the anterior segments, intraocular pressure measurement and fundus examination. A cover uncover test and an alternate cover test were performed to identify strabismus. Amblyopia was defined separately according to the age at initial presentation: < 20/50 between the ages of ≥ 3 and < 4 years, < 20/40 between the ages of ≥ 4 and < 5, < 20/30 between the ages of ≥ 5 and < 6, and < 20/25 at ages of ≥ 6. When the patients met the suggested criteria for amblyopia, as well as if there were two or more lines of decrease in visual acuity based on the above definitions, immediate cycloplegic refraction was performed and spectacle correction was planned. If there was one line of decrease in visual acuity based on the above definitions, the child was followed up, and visual acuity was checked within the next two or three months. If there was no improvement in visual acuity, cycloplegic refraction was performed and spectacles were prescribed. For cycloplegic refraction, 1% cyclopentolate was applied three times at ten-minute intervals, followed by a ten-minute waiting period before examination. When the cycloplegia was inadequate, two more drops were added at ten-minute intervals, followed by a ten-minute waiting period. After confirming that cycloplegia was adequate, refraction was performed via retinoscopic examinations by the same ophthalmologist (JWC). The success of an amblyopia treatment was defined as an improvement in visual acuity of ≥ 20/30 under the age of 6 years or of ≥ 20/25 at the age of 6 years or older. Hyperopic children with a spherical equivalent (SE) of +4.0 diopters (D) or more after cycloplegic refraction were included in this study. To investigate the course of hyperopia alone and to exclude any confounding effects of astigmatism or anisometropia, cases were excluded if astigmatism > 2.0 D or anisometropia > 2.0 D of SE was present between each eye. Additionally, cases in which there were any other ocular abnormalities or systemic diseases that affected visual acuity were excluded.

Based on the method of hyperopia correction, patients were divided into the full correction group, if the full amount of hyperopia was prescribed, or the under-correction group, if a partial amount of hyperopia was prescribed. The under-correction group was further subdivided into two subgroups based on the under-correction method. In the fixed under-correction group, the initial refractive error was reduced by a determined amount ranging from 1.0 D to 1.5 D following the recommendation of the Pediatric Eye Disease Investigator Group [23]. In the post-cycloplegic refraction (PCR) under-correction group, at one week after cycloplegic refraction, the spectacle prescription was intended to achieve the best corrected visual acuity initially with partial hyperopia correction. Therefore, the initial full correction of hyperopia was reduced by 0.25 D stepwise until no additional corrected visual acuity improvement was achieved. If constant esotropia was manifested at the initial visit, then a full correction of hyperopia was performed. If residual esotropia was observed after full correction, the patients were excluded. In all cases in the under-correction group, a cover uncover test and an alternate cover test were performed to evaluate the development of esotropia at each visit. After the spectacles were applied, each patient’s visual acuity was examined every 2 months and cycloplegic refraction was carried out every 6 months. Patients who were followed up for more than 12 months were included in this study.

### Statistical methods

To examine the differences and compare the changes between two groups, such as the full correction group and the under-correction group, or to compare changes among the three groups, such as the full correction group, the fixed under-correction group and the PCR under-correction group, a Mann-Whitney test and a Kruskal-Wallis test were used. To investigate the amount of under-correction and the decrease in hyperopia, a linear regression analysis was performed using SPSS software (version 18.0, SPSS Inc., Chicago, IL). All data for each group are presented as the mean ± standard deviation. A value of P < 0.05 was considered statistically significant.

## Results

**Fig 1:**
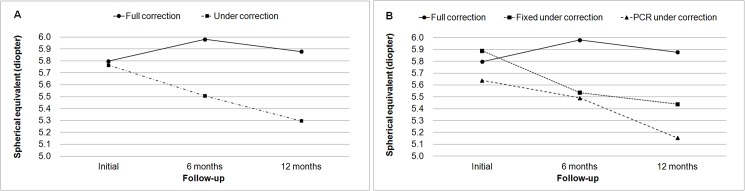
Changes in hyperopia and the progression of emmetropization in the full correction and under-correction groups. (A) Initial hyperopia was significantly reduced in the under-correction group during follow-up (P < 0.001). (B) The amount of hyperopia under-correction was correlated with the amount of hyperopia reduction (P = 0.010).

**Fig 2:**
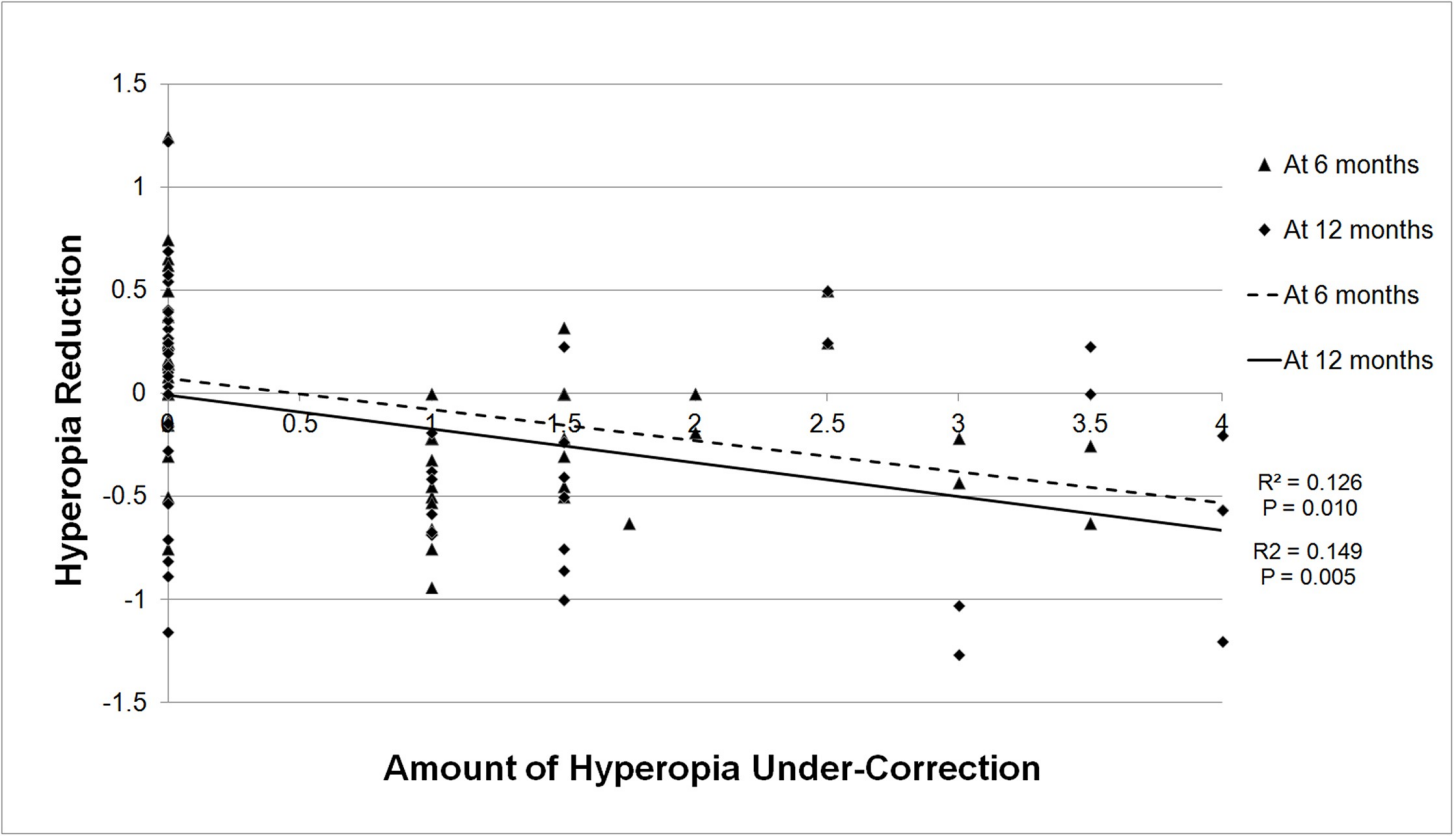
Correlation between the amount of hyperopia under-correction and hyperopia reduction.

The seventy-six eyes of thirty-eight patients who met the inclusion criteria were analyzed in this study. Mean initial cycloplegic refraction and spectacle correction were performed at 66.5 months of age, and the mean follow-up period was 30.5 months. Mean SE at the initial visit was 5.78 D (4.0–11.25 D; Table 1). There was no significant difference between the full correction group and the under-correction group. However, when comparing the 3 groups, the age of the PCR under-correction group at the initial visit was greater than those of the other groups. In the under-correction group, spectacles were prescribed with a reduction of 1.90 D. The amounts of spectacle reduction in the fixed under-correction group and the PCR under-correction group were 1.17 D and 2.62 D, respectively (Table 2). The improvement in visual acuity and the duration of amblyopia treatment did not differ between the full correction group and the under-correction group (P = 0.570, Mann-Whitney test). Improvements were achieved in 5.5 months and 5.9 months in the full correction group and the under-correction group, respectively. However, when comparing the 3 groups after dividing the under-correction group, the PCR under-correction group achieved successful amblyopia treatment faster than the other groups, reaching an improvement in 2.9 months (P = 0.001, Kruskal-Wallis test). Additionally, a decrease in hyperopia was observed during the follow-up period. At 6 months and 12 months after initial cycloplegic refraction, SE decreases of 0.07 D and 0.17 D, respectively, were observed. There was a significant decrease in hyperopia in the under-correction group compared with the decrease in the full correction group at the 6-month and 12-month examinations (P < 0.001, Mann-Whitney test). However, in the under-correction group, there was no significant difference in hyperopia reduction between the fixed and the PCR under-correction groups at the 6-month and 12-month examinations (P = 0.070 and P = 0.755, respectively, Fig 1). However, the amount of hyperopia under-correction was correlated with the amount of hyperopia reduction at the 6-month and 12-month examinations (R^2^ = 0.126, P = 0.010 at 6 months, R^2^ = 0.149, P = 0.005 at 12 months, Fig 2).

**Table 1:** The demographic characteristics of the patients.

	Full correction group	Under-correction group	P Value	Under-correction group		P value	Total
				Fixed under-correction group	PCR under-correction group		
Eyes	40	36		18	18		76
Age at initial visit (months)	61.8 ± 14.8 (43–92)	71.7 ± 25.2 (42–132)	0.114	64.2 ± 22.5 (42–107)	79.2 ± 26.0 (56–132)	0.015	66. 5 ± 20.8 (42–132)
Initial spherical equivalent	5.80 ± 2.20 (4.00–11.25)	5.76 ± 1.34 (4.00–8.50)	0.515	5.89 ± 1.39 (4.00–7.50)	5.64 ± 1.32 (4.00–8.50)	0.624	5.78 ± 1.83 (4.00–11.25)
Initial astigmatism	0.88 ± 0.70 (0–2.00)	0.92 ± 0.63 (0–2.00)	0.650	1.00 ± 0.42 (0.5–2.00)	0.83 ± 0.79 (0.00–2.00)	0.317	0.90 ± 0.66 (0.00–2.00)
Initial visual acuity (logMAR)	20/61 (20/400-20/32)	20/56 (20/200-20/32)	0.809	20/65 (20/167-20/32)	20/60 (20/200-20/32)	0.153	20/58 (20/400-20/32)
Follow-up period (months)	27.1 ± 20.5 (12–70)	34.2 ± 27.9 (12–127)	0.288	28.1 ± 13.8 (12–47)	40.4 ± 36.5 (12–127)	0.535	30.5 ± 24.4 (12–127)

PCR = post-cycloplegic refraction.

**Table 2:** The amount of hyperopia reduction with spectacle correction in the under-correction group.

	Total under-correction group	Fixed under-correction group	PCR under-correction group	P value
Mean	1.90 ± 1.03 D	1.17 ± 0.24 D	2.62 ± 1.01 D	< 0.001
Range	1.0–4.0 D	1.0–1.5 D	1.5–4.0 D	

PCR = post-cycloplegic refraction.

## Discussion

The under-correction of hyperopia in children without strabismus has shown benefits compared with the full correction. In the present study, the under-correction group showed a more rapid improvement in visual acuity and a greater reduction of hyperopia than the full correction group. Therefore, the under-correction of hyperopia can promote emmetropization even in childhood, and if the under-correction is performed safely, more emmetropization can be expected.

The incidence of hyperopia corresponding to an SE of more than +4.0 D is less than 1% [24–26]. In addition, uncorrected hyperopia of more than +4.0 D has a significant relationship with the degradation of visual acuity [25,26]. Atkinson et al. [1] demonstrated that children with hyperopia over +3.5 D in at least one meridian have a 6 times greater risk of developing amblyopia and a 13 times greater risk of developing strabismus. The authors also reported that partial spectacle correction of hyperopia could reduce the risk ratios of amblyopia and strabismus to 2.5:1 and 4:1, respectively. Although such uncorrected hyperopia correlates with a high risk of vision development impairment and although some of these patients require spectacle correction, guidelines for prescribing spectacles for hyperopic children differ, and most guidelines are based on the experiences and surveys of practitioners [27,28]. Based on a review of the literature, the American Academy of Pediatric Ophthalmology and Strabismus Vision Screening Committee has proposed standard risk factors for amblyopia that should be detected in preschool vision screening [29,30], but this committee has not suggested a protocol for prescribing spectacles for hyperopia.

Previous studies on the correction of hyperopia have focused on the success rate of amblyopia treatment or on the correlation with the development of strabismus. In contrast, in the present study, the speed of improvements of visual acuity in children with amblyopia and the progression of emmetropization were evaluated according to the method of hyperopia correction. Moreover, to confirm the correlation between the amount of hyperopia under-correction and the amount of progression of emmetropization, a greater amount of conventional under-correction was employed. In the fixed under-correction group, the amount of under-correction did not exceed 1.5 D, based on the recommendation of the Pediatric Eye Disease Investigator Group [23]. However, in the PCR under-correction group, in which an under-correction of more than 1.5 D was employed, more caution was needed because there is a greater risk of developing strabismus. MacEwen et al.[31] demonstrated that under-correction in fully accommodative esotropia can increase esotropia and that affected children can decompensate to manifest esotropia. Therefore, in the current study, the children in the PCR under-correction group were recruited if strabismus was not observed at the initial visit to avoid the development of strabismus. PCR under-correction was performed after one week of cycloplegic refraction to determine the partial amount of under-correction of hyperopia required to achieve the best corrected vision without inducing strabismus. Although there was still a risk of developing strabismus during the follow-up period, none of the children in this group developed strabismus. In the fixed under-correction group, an under-correction of 1.17 D (1.0–1.5 D) was employed, and in the PCR under-correction group, an under-correction of 2.62 D (1.5–4.0 D) was safely achieved. The demographic characteristics of the children in the different groups were not significantly different, except for their age at the initial visit: the PCR under-correction group was older than the other groups. However, this difference did not seem to influence the results because an older age correlates with disadvantages in terms of improvements in visual acuity and changes in hyperopia.

There were several advantages to using under-correction in treating moderate to severe hyperopia. First, children achieved normal visual acuity more rapidly. Although there was no difference in the duration of amblyopia treatment, the children achieved normal visual acuity more quickly after PCR under-correction because the minimum amount of under-correction required for the best corrected vision was applied in this group. Second, the children were more comfortable with spectacles that provided under-correction, and therefore, compliance was higher. Third, hyperopia showed a greater decrease, which is considered to indicate the promotion of emmetropization. There was no difference in hyperopia reduction between the fixed and the PCR under-correction groups. However, there was a negative correlation between the amount of hyperopia under-correction and hyperopia reduction (R^2^ = 0.126, P = 0.010 at 6 months, R^2^ = 0.149, P = 0.005 at 12 months, Fig 2). This result indicates that hyperopia under-correction can induce emmetropization even in childhood and that the amount of under-correction may correlate with the amount of emmetropization. These findings are compatible with the results of previous animal experiments showing that negative lens induced hyperopic defocus during the neonatal period actively regulated ocular growth and refraction to compensate for hyperopia [32,33]. This active regulation of induced defocus also appeared in myopic defocus. In myopic defocus with positive lenses, ocular growth was inhibited and myopia was decreased [34]. Based on those results, other studies have revealed that the under-correction of myopia slows the progression of myopia in children [35–37], although other studies had shown conflicting results [38,39]. The exact mechanism by which hyperopia under-correction induces emmetropization in childhood is unclear. However, in the present study, the children with moderate to severe amblyopia might not have experienced normal emmetropization process due to a greater degree of hyperopia at an earlier period and may have failed to move towards the target refractive error, emmetropia or mild hyperopia. As a result, these children could not achieve normal visual development and developed amblyopia. The initial mean refractive error was approximately 5.8 D in the full correction group and under-correction group, without a significant difference. After spectacle correction, there was no change in hyperopia in the full correction group, whereas there was a significant reduction in hyperopia in the under-correction group. Therefore, the under-correction of hyperopia not only aids in treating amblyopia but also promotes emmetropization process.

The exact mechanism by which the under-correction of moderate to severe hyperopic amblyopia promotes emmetropization process in childhood is not known. One possibility is that accommodation promotes emmetropization. Emmetropization in hyperopia is considered to progress by not only optical defocusing and blurring but also accommodation [40]. Even if optical blurring and defocusing occur before optical correction, accommodation is not activated in uncorrected states, and this status may be responsible for amblyopia. Therefore, prior to spectacle correction, the children with moderate to severe hyperopic amblyopia in the present study might exhibit only optical defocusing and blurring, and they did not undergo accommodation because it was out of range. However, after wearing spectacles, hyperopia under-correction stimulated accommodation and resulted in a recovery of emmetropization. In contrast, with the full correction of hyperopia, accommodation was not activated, and emmetropization did not progress. Among the various types of accommodation [41], tonic accommodation may have been responsible for emmetropization in this study [42]. This type refers to baseline accommodation of the resting state in hyperopia and is activated to compensate for sustained accommodative demands to overcome ongoing hyperopia but does not induce convergence [41,42]. Therefore, a remnant hyperopia of more than 1.5 D have not produced esotropia. Another possible mechanism is the re-activation of the visual feedback control system in that period. Children with moderate to severe hyperopia are thought to have failed to undergo emmetropization process at an earlier developmental stage due to unknown causes. The under-correction of hyperopia could re-activate the visual feedback control system and promote emmetropization. However, the exact mechanism by which the visual feedback control system could be re-activated during childhood after spectacle correction is unclear. Therefore, further studies should be conducted.

There were some limitations in this study. First, due to the study’s retrospective design, the patients were not randomized. Although there was no significant difference in the demographic characteristics of each group, there could have been a bias when determining hyperopia correction methods. Second, even though the progression of emmetropization in childhood was identified in the hyperopia under-correction group, the exact mechanism was not verified.

## Conclusion

Based on this study, the under-correction of moderate to severe hyperopic amblyopia has beneficial effects for treating amblyopia and activating emmetropization. In particular, PCR under-correction can improve visual acuity quickly, while both fixed and PCR under-correction can induce emmetropization and effectively reduce initial hyperopia. Therefore, although PCR under-correction is time-consuming, it is safe and effective for the treatment of moderate to severe hyperopic amblyopia. However, because there is a risk of developing esotropia during the under-correction period, one should exercise caution in selecting subjects for under-correction. The possible development of esotropia and the decompensation of binocularity should also be carefully monitored.

## Supporting information

S1 File (XLSX)
